# Poor developmental conditions decrease adult body size and egg size, but not egg laying rate and survival throughout adulthood: A long‐term experiment in a precocial bird

**DOI:** 10.1111/1365-2656.70043

**Published:** 2025-04-10

**Authors:** Oscar Vedder, Matteo Beccardi

**Affiliations:** ^1^ Institute of Avian Research Wilhelmshaven Germany

**Keywords:** ageing, birds, cascading effect, intergenerational effect, lifespan, maternal effect, silver spoon effect

## Abstract

1. The quality of the environment during individual development is generally considered to have long‐lasting effects on performance in adulthood, but this is mainly based on observational studies that cannot pinpoint the causal pathways behind such long‐term effects.
2. In this study, we performed a randomized controlled trial to test for causal effects of a poor rearing diet on performance in growth, reproduction, and survival over the complete life course of female Japanese quail (*Coturnix japonica*). All individuals were housed under standardized conditions as adults to allow separating effects of the developmental environment from effects of the adult environment.
3. The poor rearing diet led to a dramatically reduced growth, which delayed the onset of reproduction and resulted in a reduced body size throughout adulthood, as compared to a standard rearing diet. While there were no detectable effects on age‐specific egg laying rate and survival, despite strong senescence in these fitness traits, females reared with the poor diet did lay smaller eggs.
4. Hence, although there was no effect of the poor developmental environment on female laying rate and survival per se, the developmental environment of a mother did affect her adult size and the environment she provides for her offspring during embryonic development.
5. We suggest that the effects on female adult size and egg size may cause ‘silver spoon’ effects in the wild, if larger size provides an advantage in competition over resources. However, we cannot rule out that smaller size would lead to lower food requirements, thereby acting as a ‘predictive adaptive response’ to a poor environment.

## Graphical abstract

This research experimentally manipulated the quality of early‐life conditions in Japanese quail, and monitored individual performance over the complete adult lifespan under standardized conditions. It found that poor early‐life conditions decreased adult size and the size of the eggs that females lay, but did not lead to a reduced reproductive rate and a shorter lifespan per se. These results suggest that ‘silver spoon’ effects may be primarily mediated by size, and therefore would be limited to environments where size provides a competitive advantage.

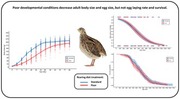

## 1 INTRODUCTION

Poor conditions during individual development are generally considered to have long‐lasting negative effects on performance in adulthood, across taxa, which can also manifest as increased senescence late in life (e.g., Cooper & Kruuk, 2018; Hamel et al., 2009; Hayward et al., 2013; Lummaa & Clutton‐Brock, 2002; Nussey et al., 2007; Reid et al., 2003; van de Pol et al., 2006; Victora et al., 2008). Yet, because assessing such long‐term effects (often referred to as ‘silver spoon’ effects; Grafen, 1988) requires long‐term studies, this general view is mainly based on correlative studies that were not a priori designed to specifically pinpoint the causal pathway that led to positive correlations between the quality of developmental conditions and late‐life performance.

From a life‐history perspective, positive correlations between the quality of the developmental environment and performance in adulthood can arise due to multiple processes. Positive feedback loops may create a process in which a poor state of an individual in one life stage directly causes a poor state at the next stage (Fokkema et al., 2021). When there is competition for resources, falling behind during development may limit the competitive abilities of an individual, causing that individual to fall even further behind and, if surviving to independence, settle in a suboptimal environment and consequently achieve lower reproductive success or adult survival (Fokkema et al., 2021; van de Pol et al., 2006; Verhulst et al., 1997). For example, in roe deer (*Capreolus capreolus*) the reduced adult fitness of late‐born fawns could be predominantly explained by their lower body mass throughout life (Plard et al., 2015).

At the same time, resource limitation may cause within‐individual trade‐offs between current performance and future performance (e.g., Lemaître et al., 2015; Metcalfe & Monaghan, 2001). When allocation to growth comes at the expense of allocation to traits that are more important later in life (e.g., DNA repair), individuals that face resource shortages during development may strategically allocate their resources to bear the brunt of the fitness costs late in life (Kirkwood, 1977). Short‐term experimental studies that manipulated developmental conditions and assessed their effects on physiological proxies of long‐term somatic quality, like telomere length, indeed suggest this to be a frequent phenomenon (Chatelain et al., 2020; Monaghan, 2014). However, assuming long‐term fitness consequences on the basis of such results may be inappropriate (Froy et al., 2021; Vedder, Moiron, et al., 2022). Hence, experimental studies that actually measure long‐term performance, in terms of fitness, are required to show whether poor developmental conditions have long‐term fitness consequences due to within‐individual trade‐offs.

Moreover, the ‘predictive adaptive response’ hypothesis suggests that poor conditions experienced during development adaptively program an individual's metabolism to cope with similar conditions as an adult (Gluckman & Hanson, 2004). However, if poor developmental conditions are not matched with poor adult conditions, this may lead to a reduced health with repercussions for fitness in adulthood (Rickard & Lummaa, 2007). Although the assumptions that are required for this mechanism to evolve are criticized, most notably on the aspect of developmental conditions providing an accurate forecast of future conditions (e.g., Nettle et al., 2013; Rickard & Lummaa, 2007; Wells, 2007), this hypothesis may similarly provide an explanation for negative effects of poor developmental conditions on performance in an unmatched adult environment (Monaghan, 2008).

Although the abovementioned pathways for long‐term fitness consequence of a poor developmental environment may not be mutually exclusive, they differ with respect to the importance of the adult environment. While experimental manipulation of the developmental environment can establish a causal effect of the manipulated variable (e.g., Spagopoulou et al., 2020), the adult environment should not be correlated with the developmental environment to rule out the possibility that the long‐term effect of the developmental environment only acts by determining the quality of the adult environment, instead of by a direct effect via within‐individual trade‐offs or a mismatched ‘predictive adaptive response’. In this respect, studies on animals in captivity have the benefit that they can more easily manipulate the developmental environment independent of the future adult environment of an individual. However, performing such experiments in a vertebrate species in which all components of fitness can be measured at the individual level throughout the complete lifespan of all individuals subjected to the experiment, with and adequate sample size, is logistically challenging.

The Japanese quail (*Coturnix japonica*) is a small galliform bird species that is ideally suitable for such an experiment. Chicks are precocial and can be easily raised independent of the parents, allowing for manipulation of the quality of the developmental environment without any confounding variables. For a bird, they have an extremely fast life history, with individuals being able to achieve reproduction within 2 months after hatching (Vedder, Bichet, et al., 2022). Moreover, because domesticated quail have been primarily selected for egg‐laying performance (Lukanov & Pavlova, 2020) their ‘fitness’ (that is, what is maximized by selection) and reproductive performance can be easily quantified by their survival and the number and size of the eggs they lay (Vedder, Bichet, et al., 2022). Senescence in survival and egg laying starts almost immediately after the onset of laying, and their lifespan rarely exceeds 3 years (Beccardi et al., 2024; Vedder, Bichet, et al., 2022; Woodard & Abplanalp, 1971), allowing for lifelong monitoring of performance and senescence rates within a foreseeable time frame.

In this study we experimentally manipulated the postnatal developmental conditions of Japanese quail chicks until sexual maturity, by randomly providing them with either a standard or a poor quality rearing diet (see also Vedder et al., 2023). From these chicks, 218 females were subsequently kept communally in a single environment until they died naturally. We monitored all components of fitness (onset of reproduction, daily survival, and age‐specific fecundity), per capita allocation to reproduction (fresh egg mass), and adult size and age‐specific body mass over their complete lifespans. This allowed us to test if the poor conditions during development had long‐lasting effects on adult performance or senescence rates, independent of the adult environment, and whether this had persisting effects on body mass throughout adulthood.

## 2 METHODS

### 2.1 Breeding

To acquire the chicks for the experiment, we bred with ca. 1‐year‐old adult Japanese quail in March–June of 2019 and 2020 at the Institute of Avian Research in Wilhelmshaven, Germany. In both years, we performed four rounds of breeding, with a total of 96 pairs in 2019 and a total of 80 pairs in 2020. Per round, pairs were housed in breeding cages in which they spent 10–14 days together. At this age, females lay at a rate of ca. 0.8 eggs per day (Vedder, Bichet, et al., 2022), and we collected and marked all eggs on a daily basis. Males readily copulate when placed together with a female, causing most eggs to already be fertile after the pair spent 2 days in the breeding cages (Vedder, 2022). Before pairing, females were housed together and isolated from males for at least fourteen days to prevent stored sperm of previous males from fertilizing any eggs (see Birkhead & Fletcher, 1994). The collected eggs were stored at 12°C and artificially incubated per round, always within 14 days of laying. Incubation was performed at 37.7°C and 50% relative humidity with fully automatic incubators (Grumbach, ProCon automatic systems GmbH & Co. KG, Mücke, Germany). The eggs were automatically turned every hour for the first 14 days of incubation. After 14 days of incubation, eggs were placed in marked individual compartments to allow the hatchling to be linked to the egg it hatched from. Subsequent incubation was performed at 37.2°C and 70% relative humidity, without egg turning, in hatching incubators (Favorit, HEKA Brutgeräte, Rietberg, Germany). After 16 days of incubation, the hatching incubators were checked once a day for new hatchlings until no viable eggs were left (after ca. 19 days of incubation).

### 2.2 Experimental procedure and general data collection

After hatching, all chicks were marked with a numbered plastic leg ring and weighed to the nearest 0.01 g. They were randomly distributed over heated rearing cages (109 × 57 × 25 cm, Kükenaufzuchtbox Nr 4002/C, HEKA Brutgeräte, Rietberg, Germany) with one of two rearing diets differing in quality, in such a way that offspring from the same pair were roughly equally divided over the two diet treatments. In total, we used 8 rearing cages (4 per treatment), with a maximum of 30 chicks of both sexes combined simultaneously in the same cage. We used a standard poultry rearing diet with 21.0% protein, 4.0% fat, and 1.1% calcium as the ‘standard’ rearing treatment. For the ‘poor’ rearing treatment, we used a diet containing 14.5% protein, 4.0% fat, and 1.0% calcium (see Online Supporting Information for a detailed overview of both diets). Both diets had the same caloric value of 11.4 MJ/Kg and are commercially available poultry feeds (GoldDott, DERBY Spezialfutter GmbH, Münster, Germany), yet the standard one is intended for growing chicks, while the ‘poor’ one is intended for fully‐feathered, but not yet laying, hens. The lower protein content of the ‘poor’ diet was anticipated to substantially reduce growth rate without compromising chick survival (Weber & Reid, 1967). The protein content of the standard diet was anticipated to result in a normal growth rate without leading to impairments associated with too rapid growth (Weber & Reid, 1967). Food and water were provided ad libitum, and the food was ground to a homogeneous powder to prevent selective eating. The temperature of all rearing cages was set at 37.0°C at hatching and gradually lowered to 20–25°C over the course of 3 weeks. They were kept on a 16–8 h light–dark cycle for both treatments, and their plastic leg rings were replaced with uniquely numbered aluminium rings when chicks were between 14 and 35 days old, depending on the size of their legs. All chicks were transferred to outdoor aviaries when they were 21 days old. For this, we used two separate but identical aviaries, in which they were kept on the same rearing diets as they were on before. These aviaries were equipped with heat lamps and received a minimum of 16 h of light.

Chick mortality was low (5.7%), mostly occurred in the first few days after hatching, and did not differ between the diet treatments (Vedder et al., 2023). All surviving chicks were weighed at weekly intervals after their individual hatching date, to the nearest 0.01 g at 7 days, and to the nearest g at all subsequent ages until 84 days old when all individuals were full‐grown. At 84 days also the full‐grown tarsus of each individual was measured, to the nearest 0.01 mm. We sexed all chicks based on plumage characteristics after about 28 days old. While the male chicks were also monitored until 84 days old as part of another study (Vedder et al., 2023), in this study we hereafter only focus on the females because only their fecundity was monitored over their entire lifespan. From 35 days old onwards, they were individually checked for the onset of egg laying every 2–3 days. This was done by checking for the presence of an egg in the oviduct through palpation. At the onset of egg laying, each female was housed in a breeding cage for a week. In these cages, they all received an adult layer diet, with 19.0% protein, 4.6% fat, and 4.8% calcium, and a caloric value of 9.8 MJ/Kg (GoldDott, DERBY Spezialfutter GmbH, Münster, Germany). This way, individual laying rates were established by checking for the presence of a newly laid egg every day for 7 days in a row. All new eggs were collected and weighed to the nearest 0.01 g within a day of laying. Afterwards, in 2019 all females (*n* = 151) of both treatment groups were moved to a single outdoor aviary in which all received the same adult layer diet (ad libitum provided by multiple feeders placed throughout the aviary), with a minimum of 16 h of light per day to ensure the continuation of reproductive activity (Kovach, 1974). In 2020, half of all females were also housed under those conditions as adults (*n* = 66), while the other half was housed under different conditions in winter as part of another experiment and not included in this study. The excluded females were randomly selected at the individual level (using software to generate a random number per individual, and a cut‐off value chosen to exclude half of the sexually mature females). In 2019, three females never started laying (1 with the standard diet, and 2 with the poor diet) and they were moved to the aviary with the adult diet when completely full grown and remained part of the study. All adult mortality was monitored on a daily basis, and all females were weighed and temporarily monitored for reproductive performance as described above (laying rate and egg mass across 7 days) at approximately 0.5‐year intervals over their complete lifespans. The study did not require any invasive sampling, and all procedures involving the quail were done under licence of the “Veterinäramt JadeWeser” (permit nr. 42508_03122020).

### 2.3 Statistical analyses

Although we have previously reported a large effect of the diet manipulation on postnatal growth for all individuals of both sexes (Vedder et al., 2023), we here tested the effect for the subset of females that were housed as adults under the same conditions (*n* = 217), of which 105 (48.4%) had received the poor diet, and 112 (51.6%) the standard diet. To this end we used a Generalized additive model (GAM, function ‘gam()’, package ‘mgcv’), with a normal error distribution, with all weekly body mass measurements as dependent variable. To test if growth differed between both diet treatments, we added rearing diet treatment (two levels: standard/poor) as fixed effect and its interaction with age as a smoothed variable. We also controlled for potential differences between years, by adding year (two levels: 2019/2020) as a fixed effect. As random effects we added parent ‘pair identity’, and ‘individual identity’, to account for non‐independence of full siblings, and repeated measures of the same individuals. While a GAM has the advantage that it does not constrain the growth curve to a specific shape, it has the disadvantage that it does not allow for straightforward comparison of specific growth parameters with other studies. We therefore also tested which commonly used growth function (Gompertz, logistic, log‐logistic, Weibull) best fitted the observed growth (with the ‘drm()’ function from the R‐package ‘drc’) and provide a comparison of growth parameters between the diets for the best fitting function.

For the dependent variables that were only measured once per individual (adult tarsus length, age of sexual maturity, and adult lifespan) we applied three separate Linear mixed models (LMMs, function ‘lmer()’, package ‘lme4’), to test the effect of the rearing diet manipulation. In these models, diet treatment and hatching year were included as fixed effects, and parent ‘pair identity’ as random effect.

To test for age‐specific differences in adult body mass, and egg mass, between the two rearing diets we performed two separate GAMs with a normal error distribution and body mass, and egg mass, as dependent variables, respectively. These models included rearing diet treatment as fixed effect and its interaction with age as a smoothed variable. Also in this case, we controlled for potential differences between cohorts, by including hatching year as a fixed effect. In addition, ‘pair identity’ and ‘individual identity’ were included as random effects. Because we were specifically interested in differences in within‐individual body mass/egg mass patterns with age, we accounted for potential selective mortality in relation to adult body mass/egg mass by including adult lifespan as a continuous linear predictor (following van de Pol & Verhulst, 2006). However, if diet has a similar effect on lifespan as on adult body mass or egg mass, correcting for lifespan may mask such effects. We therefore centred the lifespan variable around the mean lifespan per diet treatment (by subtracting the mean from the raw value), such that there was no overall difference in lifespan between the diet treatments. Moreover, we repeated the model on egg mass with adult body mass during laying added as a smoothed variable, to test if there was also a rearing diet effect on egg mass when correcting for differences in adult body mass.

The effect of the rearing diet manipulation on daily laying probability was tested with a GAM with a binomial error distribution and a logit link function. For this analysis the dependent variable consisted of 7 binomial records (1 = egg laid, 0 = no egg laid) per age‐specific sampling period. Diet treatment was added as fixed effect and we specifically included the interaction term with age as a smoothed variable to test whether the poor rearing diet could accelerate reproductive senescence. Again, we included hatching year as a fixed effect, and ‘pair identity’ and ‘individual identity’ as random effects. Adult lifespan (mean‐centred per diet) was included as a continuous linear predictor, to specifically test for within‐individual effects of the diet treatment.

Finally, to study the effect of the diet manipulation on their daily adult mortality rate we used a piece‐wise exponential additive mixed model (PAMM), combining the ‘pammtools’ package (Bender et al., 2018) with a GAM model fitted with the ‘mgcv’ package. The model included diet treatment and hatching year as fixed factors, the interaction between diet treatment and age (smoothed variable) as interaction term, and ‘pair identity’ as random effect. In this case, a Poisson error distribution was applied. Although this approach is recommended for age‐specific survival data that does not follow a preconceived shape with age (Bender et al., 2018), we also tested for a difference in age‐specific survival between the diet treatments with a parametric survival model. Because we have previously shown that the Weibull function provides the best fit for Japanese quail survival data (Beccardi et al., 2024), we ran a model in which we tested for differences in the Weibull function's scale (baseline mortality) and shape (age‐specific mortality) parameter between the diets, with the ‘flexsurvreg()’ function from R‐package ‘flexsurv’. This model accounted for potential cohort differences in survival by allowing the scale parameter to vary with hatching year.

For all the performed GAMs, we used the gam() function's default thin plate splines with 10 knots, estimated using either a maximum likelihood (ML) approach (chick body mass, adult body mass, egg mass) or a restricted maximum likelihood (REML) approach (daily laying probability, daily adult mortality rate). Because the GAMs do not provide p‐values for interaction effects, we assessed the significance of age‐specific effects of the diet manipulation by estimating separate smoothers for the two different levels of the diet predictor and calculating the difference between the smoothers with its approximate 95% CI (function ‘difference_smooths()’, package ‘gratia’; Wood, 2017, p. 294). In the absence of an overall effect of rearing diet, this should allow us to infer that there is a difference between the rearing diets in the age range where the confidence interval does not overlap with zero (see Online Supporting Information). All analyses were done in R software (version 4.1.3, R Foundation for Statistical Computing).

## 3 RESULTS

### 3.1 Growth and adult size

**FIGURE 1:**
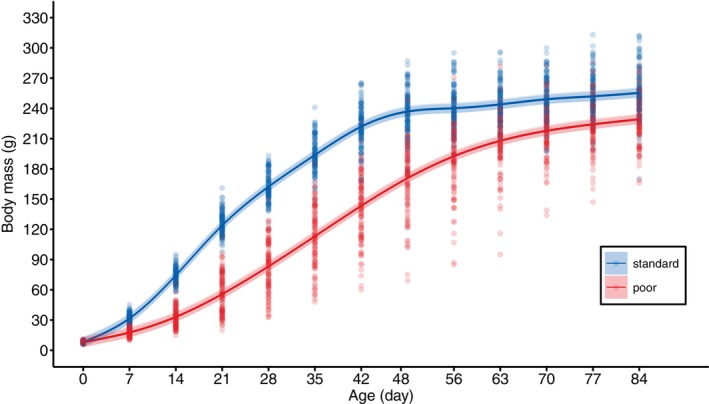
Model predicted body mass of female Japanese quail chicks, fed with either a standard (blue) or poor (red) rearing diet, in relation to age. The correspondingly coloured shaded areas represent the 95% CIs. The datapoints represent the raw data.

**FIGURE 2:**
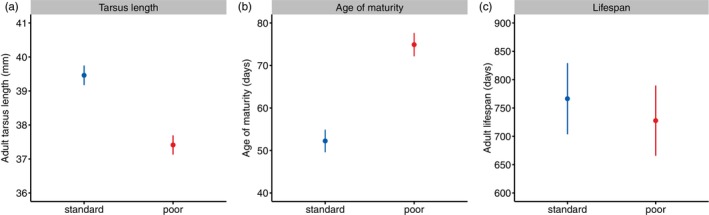
Comparison between the effect of the standard rearing diet (blue) and poor rearing diet (red) on (a) adult tarsus length, (b) age of maturity, and (c) adult lifespan. The graphs represent predicted values (mean ± 95% CI) from the LMMs presented in Table 2.

The two different rearing diets led to extreme differences in growth, with the poor diet leading to lower body mass from the first measurement after hatching, at an age of 7 days, onwards (Figure 1, Figure S1, Table 1). At peak growth, the females that received the poor diet weighed ca. 55% less than the females that received the standard diet (Figure 1). Although this difference became smaller towards the end of the growth stage, the females with the poor diet were not able to completely catch up and remained ca. 10% lighter when full‐grown (Figure 1). The length of their tarsus was ca. 5% shorter when full‐grown at 12 weeks of age (Figure 2a, Table 2).

**TABLE 1:** Summaries of GAMs testing for effects of the rearing diet manipulation on chick and adult age‐specific body mass of female Japanese quail.

Chick body mass (g)			
Parametric coefficients	Estimate	S.E.	*p*‐value
Intercept	175.748	1.958	<0.001
Diet (poor)	−45.909	2.166	<0.001
Hatching year (2020)	1.299	2.760	0.638
Smooth terms	Edf	*F*‐value	*p*‐value
Age: Diet (standard)	8.597	6281.06	<0.001
Age: Diet (poor)	7.682	5408.77	<0.001
Random effects	Variance (SD)	95% CI	*p*‐value
Pair identity	54	44.22–65.8	0.005
Individual identity	211	182.6–260.7	<0.001
Residual	188	134.0–243.1	

Adult body mass (g)			
Parametric coefficients	Estimate	S.E.	*p*‐value
Intercept	256.987	2.628	<0.001
Diet (poor)	−13.046	2.755	<0.001
Hatching year (2020)	−1.363	3.786	0.719
Lifespan	0.005	0.005	0.285
Smooth terms	Edf	*F*‐value	*p*‐value
Age: Diet (standard)	3.948	13.004	<0.001
Age: Diet (poor)	4.377	10.862	<0.001
Random effects	Variance (SD)	95% CI	*p*‐value
Pair identity	131	122.9–147.3	<0.001
Individual identity	219	206.7–236.9	<0.001
Residual	399	380.1–420.1	

**TABLE 2:** Summaries of LMMs testing for effects of the rearing diet manipulation on adult tarsus length, age of sexual maturity, and adult lifespan of female Japanese quail.

Random effects	Variance	Fixed effects	Estimate	S.E.	*p*‐value
**Tarsus length (mm)**					
Pair identity	0.400	Intercept	39.461	0.145	<0.001
		Diet (poor)	−2.048	0.152	<0.001
		Hatching Year (2020)	0.114	0.207	0.583
**Age of maturity (days)**					
Pair identity	24.820	Intercept	52.246	1.348	<0.001
		Diet (poor)	22.652	1.529	<0.001
		Hatching Year (2020)	0.920	1.904	0.630
**Lifespan (days)**					
Pair identity	8852	Intercept	727.680	31.450	<0.001
		Diet (poor)	−38.700	37.300	0.301
		Hatching Year (2020)	−128.570	44.550	0.005

The growth in body mass was best explained by a Gompertz function (Table S1), which indicated that the intrinsic growth constant (k) of females with the poor diet was ca. 37% lower, and the age of peak growth (*t*_*i*_) ca. 75% later compared to the females with the standard diet (Figure S2, Table S2). The asymptotic body mass (A) was estimated to be only ca. 2% lower for the females with the poor diet (Table S2), but the asymptote for these females was not perfectly captured by the Gompertz function (Figure S2).

**FIGURE 3:**
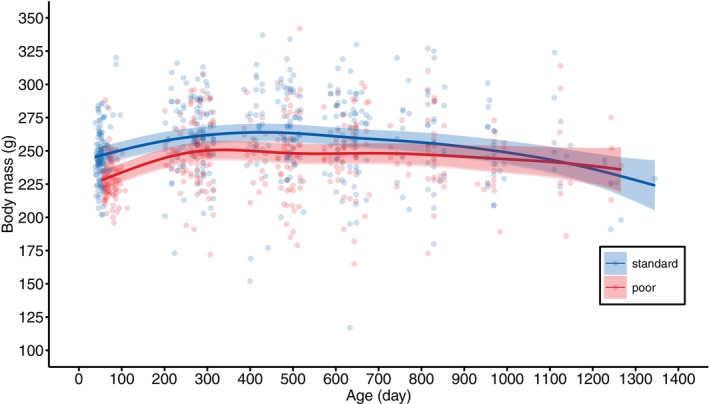
Model predicted adult body mass of female Japanese quail, reared with either a standard (blue) or poor (red) diet, in relation to age. The correspondingly coloured shaded areas represent the 95% CIs. The datapoints represent the raw data.

Indeed, overall the adult body mass of females with the poor diet was estimated to be ca. 5% lower than that of females with the standard diet, with this difference remaining detectable until ca. 2.5 years old (Figure 3, Figure S3, Table 1). After 2.5 years, the difference in body mass disappeared, but this is most likely due to only a few females reaching that age, causing increased 95% CIs at late age (Figure 3, Figure S3). There was no selective mortality with respect to body mass, because lifespan and adult body mass were unrelated (Table 1).

### 3.2 Reproduction

**FIGURE 4:**
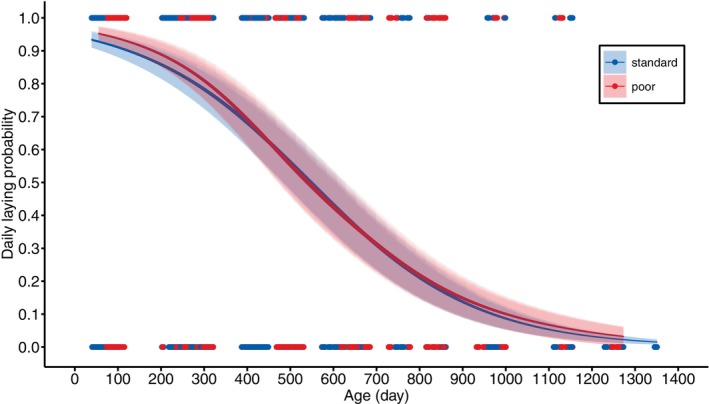
Model predicted daily laying probability of female Japanese quail, reared with either a standard (blue) or poor (red) diet, in relation to age. The correspondingly coloured shaded areas represent the 95% CIs. The datapoints represent the raw data, and indicate the ages at which individuals were sampled.

The onset of egg laying was delayed by ca. 23 days in the females that received the poor rearing diet (ca. 40% later, Figure 2b, Table 2). The laying rate was, however, not affected by the rearing diet (Figure 4, Figure S4, Table 3). Females of both diet treatments started off with laying almost an egg per day, but senesced rapidly, with three‐year‐old females laying almost no eggs anymore (Figure 4). This decline was independent of rearing diet (Figure 4, Figure S4, Table 3). Lifespan was positively related to laying rate, indicating that the most fecund females were also the most viable (Table 3).

**TABLE 3:** Summaries of GAMs testing for effects of the rearing diet manipulation on daily laying probability and egg mass (with and without correcting for adult body mass), of female Japanese quail.

Daily laying probability			
Parametric coefficients	Estimate	S.E.	*p*‐value
Intercept	0.615	0.149	<0.001
Diet (poor)	0.168	0.181	0.353
Hatching year (2020)	0.057	0.214	0.790
Lifespan	0.001	0.001	0.003
Smooth terms	Edf	Chi. sq	*p*‐value
Age: Diet (standard)	1.002	573.1	<0.001
Age: Diet (poor)	2.443	479.6	<0.001
Random effects	Variance (SD)	95% CI	*p*‐value
Pair identity	0.147	−0.236–0.530	0.703
Individual identity	1.300	0.969–1.340	<0.001

Egg mass (g)			
Parametric coefficients	Estimate	S.E.	*p*‐value
Intercept	11.578	0.113	<0.001
Diet (poor)	−0.191	0.090	0.033
Hatching year (2020)	−0.101	0.168	0.547
Lifespan	−0.001	0.001	0.256
Smooth terms	Edf	*F*‐value	*p*‐value
Age: Diet (standard)	8.487	307.78	<0.001
Age: Diet (poor)	7.360	162.40	<0.001
Random effects	Variance (SD)	95% CI	*p*‐value
Pair identity	0.463	0.356–0.580	<0.001
Individual identity	0.267	0.146–0.498	0.003
Residual	0.529	0.328–0.745	

Egg mass (g) (corrected for adult body mass)			
Parametric coefficients	Estimate	S.E.	*p*‐value
Intercept	11.497	0.105	<0.001
Diet (poor)	−0.035	0.087	0.687
Hatching year (2020)	−0.075	0.155	0.628
Lifespan	−0.002	0.001	0.198
Smooth terms	Edf	*F*‐value	*p*‐value
Age: Diet (standard)	8.287	151.58	<0.001
Age: Diet (poor)	7.191	62.96	<0.001
Body mass	5.216	13.85	<0.001
Random effects	Variance (SD)	95% CI	*p*‐value
Pair identity	0.383	0.219–0.503	<0.001
Individual identity	0.239	0.121–0.368	<0.001
Residual	0.521	0.405–0.739	

**FIGURE 5:**
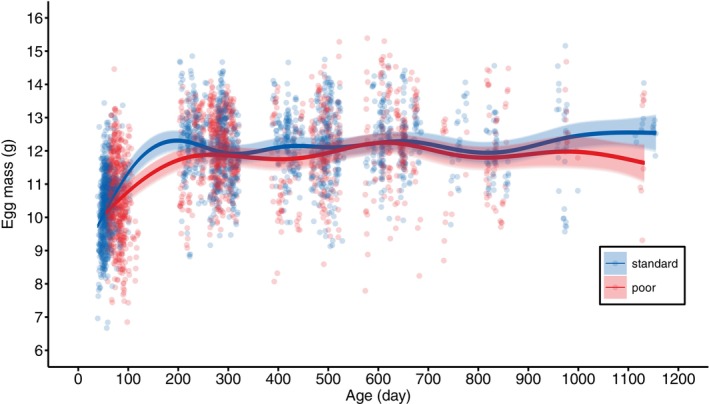
Model predicted mass of eggs laid by female Japanese quail, reared with either a standard (blue) or poor (red) diet, in relation to age. The correspondingly coloured shaded areas represent the 95% CIs. The datapoints represent the raw data.

The females that had received the poor rearing diet did lay smaller eggs (Figure 5, Table 3). This effect was not present in their first week of egg laying, when all females still lay relatively small eggs, but appeared at later ages, when females had reached their peak egg size (Figure 5, Figure S5). Egg size remained relatively constant after its peak and was not associated with lifespan (Figure 5, Table 3). The diet effect on egg size disappeared after correcting for adult body mass during laying (Table 3), suggesting that the diet effect on egg size is not independent from its effect on adult body size.

### 3.3 Survival

**FIGURE 6:**
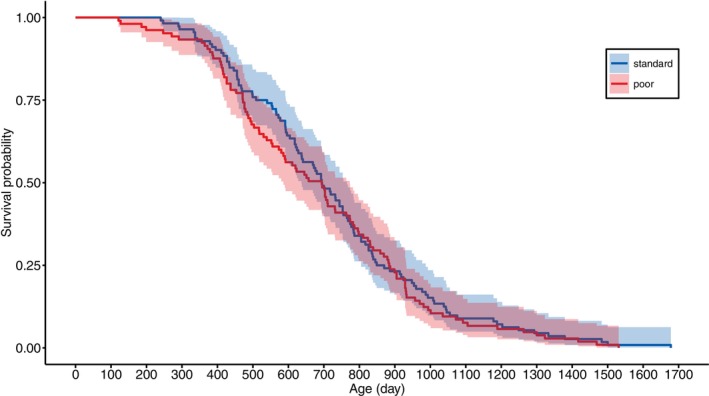
Raw survival curves of female Japanese quail, reared with either a standard (blue) or poor (red) diet. The correspondingly coloured shaded areas represent the 95% CIs.

**FIGURE 7:**
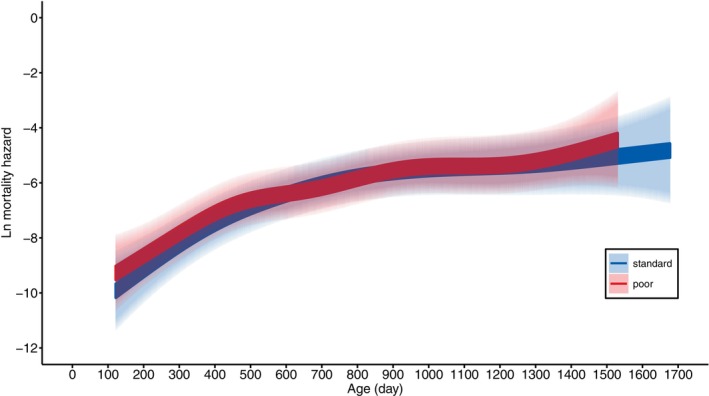
Model predicted mortality hazard of female Japanese quail, reared with either a standard (blue) or poor (red) diet, in relation to age. The correspondingly coloured shaded areas represent the 95% CIs.

The females lived, on average, for ca. 2 years, with the cohort of 2020 living shorter than the cohort of 2019 (ca. 17% shorter, Table 2). The rearing diet had no effect on lifespan (Figure 2c, Table 2). This was confirmed with an analysis of their age‐specific mortality rate (Figures 6 and 7, Table 4). Mortality increased with age, but this was independent of rearing diet (Figure 7, Figure S6, Table 4). Also, when adult mortality was modelled with a Weibull function, there was no indication that the rearing diet had an effect on mortality, as the 95% confidence interval for the effect of diet on both the scale (baseline mortality) and shape (age‐specific mortality) parameters overlapped with zero (Table S3).

**TABLE 4:** Summary of GAM testing for effects of the diet manipulation on age‐specific adult mortality rate of female Japanese quail.

Adult mortality hazard			
Parametric coefficients	Estimate	S.E.	*p*‐value
Intercept	−6.639	0.146	<0.001
Diet (poor)	0.192	0.168	0.254
Hatching year (2020)	0.535	0.183	0.003
Smooth terms	Edf	Chi. sq	*p*‐value
Age: Diet (standard)	3.711	88.08	<0.001
Age: Diet (poor)	4.131	80.29	<0.001
Random effects	Variance (SD)	95% CI	*p*‐value
Pair identity	0.171	0.093–0.313	0.027

## 4 DISCUSSION

A poor environment during individual development is generally considered to have long‐lasting negative effects on performance in adulthood (e.g., Cooper & Kruuk, 2018; Hamel et al., 2009; Hayward et al., 2013; Lummaa & Clutton‐Brock, 2002; Nussey et al., 2007; Reid et al., 2003; van de Pol et al., 2006; Victora et al., 2008). Yet, the ubiquity of such ‘silver spoon’ effects (Grafen, 1988) is questioned, as the absence of long‐term effects is also frequently reported (Cam et al., 2003; Drummond & Rodríguez, 2013; Vedder, Bichet, et al., 2022; Vedder & Bouwhuis, 2018; Wilkin & Sheldon, 2009). More knowledge on the causal pathways that lead to ‘silver spoon’ effects may help explain why the occurrence of such effects varies between studies. In this study, we performed a randomized controlled trial to test for causal effects of a poor rearing diet on performance in growth, reproduction, and survival, over the complete life course of female Japanese quail. We specifically housed all females under standardized conditions as adults, to test for effects of the developmental environment that are independent from effects of the adult environment.

Although the poor diet led to a dramatically reduced growth and resulted in a reduced body size throughout adulthood, there were no detectable effects on age‐specific reproduction (egg laying) and survival over their complete lifespan. The only notable diet treatment effect on a component of ‘fitness’ in this domesticated bird species, was the delayed onset of reproduction in females reared with the poor diet. On average, these females started laying 23 days later than the females reared with a standard diet (Table 2). Considering that egg laying rate in Japanese quail starts off high with almost an egg per day (Figure 4, Figure S7), this equates to having laid ca. 21 less eggs in this period. Since the lifetime number of eggs an average female lays can be estimated to be 395 (see Online Supporting Information), this delayed onset of reproduction already constitutes a ca. 5% reduction in ‘fitness’. This illustrates the selection for early‐life performance in this avian model species, and their suitability to experimentally study eco‐evolutionary processes that may affect performance over the complete lifespan, within a foreseeable time frame, even though no wild bird species may lay so many eggs.

Despite the absence of other effects on ‘fitness’, the result that females reared with the poor diet could not completely catch up in size and body mass in adulthood does suggest that ‘silver spoon’ effects can be caused by positive feedback loops, in which a size advantage in competition for resources would lead to positive reinforcement in access to resources (Fokkema et al., 2021). Indeed, if ‘silver spoon’ effects arise mainly due to a reduced competitiveness, or resource holding power (Parker, 1974), the absence of effects on reproduction and adult survival in benign adult conditions fits with expectation. While studies on animals in the wild have suggested this as a possible pathway behind ‘silver spoon’ effects (Plard et al., 2015; van de Pol et al., 2006; Verhulst et al., 1997), such studies cannot rule out additional mechanisms responsible for ‘silver spoon’ effects. For example, if individuals were to maintain growth and survival under poor conditions at the expense of allocation to DNA repair (e.g., Lemaître et al., 2015; Metcalfe & Monaghan, 2001), a reduced performance late in life should also be apparent under standardized adult conditions. However, as we did not find evidence for increased senescence in reproductive performance and survival in the females reared with the poor diet, such delayed effects resulting from within‐individual trade‐offs may be comparatively small.

Similarly, the ‘predictive adaptive response’ hypothesis suggests that negative long‐term effects can occur due to a mismatch between the developmental environment and the adult environment, because during development individuals may be primed for a future environment that matches the developmental environment (Gluckman & Hanson, 2004; Monaghan, 2008). Since the conditions for such a mechanism to be adaptive, and thereby able to evolve, are relatively stringent (e.g., Nettle et al., 2013; Rickard & Lummaa, 2007; Uller et al., 2013; Wells, 2007), we did not specifically set out to test this hypothesis with two different adult environments. Nevertheless, our standard adult poultry diet will most likely have better matched the standard chick diet and should have led to a reduced adult performance for the females reared with the poor diet, if the ‘predictive adaptive response’ mechanism would have been acting. The fact that we did not find evidence for this to occur may be explained by an overruling effect of the quality of the current environment. However, we cannot rule out that smaller adult size would lead to better performance in a poor adult environment by lowering food requirements, with small size acting as a ‘predictive adaptive response’ to poor food availability in adulthood (Monaghan, 2008).

The importance of the current environment is also suggested by the difference in adult survival between the two cohorts. This is most likely attributed to current environmental effects, since both cohorts were reared equally, but as adults housed in an outdoor aviary relatively unprotected against fluctuations in ambient temperature. As, alternative to the ‘predictive adaptive response’ hypothesis, it has been reported that ‘silver spoon’ effects are exacerbated in a poor adult environment (Briga et al., 2017), any ‘silver spoon’ effect that is not caused by a positive correlation between the quality of the developmental and adult environment could have been stronger in our cohort with the shortest lifespan. However, a post hoc analysis testing for an interaction effect between diet treatment and hatching year on lifespan did not detect such an effect (*p* = 0.696, Table S4). Hence, regardless of exact adult environmental conditions, our experimental approach does not provide evidence for detectable long‐term effects of a poor developmental environment per se.

Because the smaller size of the females reared with the poor diet also led to them laying smaller eggs in adulthood, our results do provide evidence for effects of a mother's developmental environment on her offspring's embryonic developmental environment. When positive effects are carried through to all of the offspring's subsequent life stages (as discussed above), this can cause cascading transgenerational effects with repercussions over generations (Aiken et al., 2015; Pick et al., 2019). However, the potential for such transgenerational effects is again most likely modulated by the environment. Previous research on Japanese quail showed a strong correlation between egg size and offspring size at hatching, but the effects of egg size were replaced by genetic and current environmental effects rapidly thereafter, when chicks were growing and food was provided ad libitum (Vedder et al., 2023). In the wild, such an initial size advantage may be more likely maintained with increased competition for food (Fokkema et al., 2021; Krist, 2011; Oddie, 2000).

In sum, we did not find evidence for long‐term effects of a poor developmental environment on adult survival and reproduction. However, because we did find persisting effects on adult size and egg size, we cannot rule out that in the wild a size advantage in competition for resources can create a positive correlation between the quality of the environment in different life stages. This would cause a ‘silver spoon’ effect, even if the quality of the current environment would be the most important environmental factor in determining individual performance. Alternatively, smaller size may lead to lower food requirements, thereby acting as a ‘predictive adaptive response’ to a poor environment.

## AUTHOR CONTRIBUTIONS

Conceptualization and experimental design: Oscar Vedder. Data collection: Oscar Vedder and Matteo Beccardi. Data analysis: Matteo Beccardi, with feedback from Oscar Vedder. Writing: Oscar Vedder, with feedback from Matteo Beccardi.

## CONFLICT OF INTEREST STATEMENT

The authors have no conflict of interest.

## ETHICS STATEMENT

The study did not require any invasive sampling, and all procedures involving the quail were done under licence of the “Veterinäramt JadeWeser” (permit nr. 42508_03122020).

## Supporting information


**Figure S1.** Model predicted difference in body mass (in g), with 95% CI, between female Japanese quail chicks fed with a standard or poor rearing diet, in relation to age.
**Figure S2.** Growth in body mass of female Japanese quail chicks fed with a standard or poor rearing diet.
**Table S1.** AIC values of growth models using four common growth functions, ranked according to goodness of fit.
**Table S2.** Parameter values of the Gompertz growth function fitted for female Japanese quail chicks fed with a standard or poor rearing diet.
**Table S3.** Summary of a Weibull mortality model testing for effects of the rearing diet on age‐specific adult mortality hazard of female Japanese quail.
**Table S4.** Summary of a LMM testing for an interaction effect between rearing diet and hatching year on adult lifespan (in days) of female Japanese quail.
**Figure S3.** Model predicted difference in adult body mass (in g), with 95% CI, between female Japanese quail reared with a standard or poor diet, in relation to age.
**Figure S4.** Model predicted relative difference (odds ratio), with 95% CI, in daily laying probability of female Japanese quail reared with a standard or poor diet. The dashed line represents the odds ratio for which there is no difference between the standard and poor rearing diet.
**Figure S5.** Model predicted difference (in g), with 95% CI, in mass of eggs laid by female Japanese quail reared with a standard or poor diet, in relation to age.
**Figure S6.** Model predicted relative difference (odds ratio), with 95% CI, in adult mortality hazard of female Japanese quail reared with a standard or poor diet, in relation to age.
**Figure S7.** (A) Model predicted daily laying probability of female Japanese quail in relation to age, with 95% CI (shaded). The datapoints represent the raw data, and indicate the ages at which individuals were sampled. (B) Observed probability of an adult female to reach a certain age, with 95% CI (shaded).

## Data Availability

Data available from the Dryad Digital Repository https://doi.org/10.5061/dryad.0gb5mkmc7 (Vedder & Beccardi, 2025).
