# Supplementary material for: Poor developmental conditions decrease adult body size and egg size, but not egg laying rate and survival throughout adulthood: A long‐term experiment in a precocial bird
Source: J Anim Ecol. 2025 Apr 10;94(6):1231–43. doi: 10.1111/1365-2656.70043 (PMC12134424; doi:10.1111/1365-2656.70043)
Supplement: Supplementary file 1 — Figure S1. Model predicted difference in body mass (in g), with 95% CI, between female Japanese quail chicks fed with a standard or poor rearing diet, in relation to age. Figure S2. Growth in body mass of female Japanese quail chicks fed with a standard or poor rearing diet. Table S1. AIC values of growth models using four common growth functions, ranked according to goodness of fit. Table S2. Parameter values of the Gompertz growth function fitted for female Japanese quail chicks fed with a standard or poor rearing diet. Table S3. Summary of a Weibull mortality model testing for effects of the rearing diet on age‐specific adult mortality hazard of female Japanese quail. Table S4. Summary of a LMM testing for an interaction effect between rearing diet and hatching year on adult lifespan (in days) of female Japanese quail. Figure S3. Model predicted difference in adult body mass (in g), with 95% CI, between female Japanese quail reared with a standard or poor diet, in relation to age. Figure S4. Model predicted relative difference (odds ratio), with 95% CI, in daily laying probability of female Japanese quail reared with a standard or poor diet. The dashed line represents the odds ratio for which there is no difference between the standard and poor rearing diet. Figure S5. Model predicted difference (in g), with 95% CI, in mass of eggs laid by female Japanese quail reared with a standard or poor diet, in relation to age. Figure S6. Model predicted relative difference (odds ratio), with 95% CI, in adult mortality hazard of female Japanese quail reared with a standard or poor diet, in relation to age. Figure S7. (A) Model predicted daily laying probability of female Japanese quail in relation to age, with 95% CI (shaded). The datapoints represent the raw data, and indicate the ages at which individuals were sampled. (B) Observed probability of an adult female to reach a certain age, with 95% CI (shaded). [file JANE-94-1231-s001.docx]

**Poor developmental conditions decrease adult body size and egg size, but not egg laying rate and survival throughout adulthood: a long-term experiment in a precocial bird.**

**—Online Supporting Information—**

**Oscar Vedder & Matteo Beccardi**

**Detailed description of the two rearing diets**

**Standard diet:**

**Ingredients:** Crude protein 21.0%, crude fat 4.0%, crude fibre 3.5%, crude ash 7.0%, lysine 1.1%, methionine 0.45%, calcium 1.1%, phosphorus 0.7%, sodium 0.15%, energy 11.4 MJ ME/kg.

**Additives per kg:**

**Nutritional additives:** 8,600 i.U. Vitamin A (3a672a), 3.420 i.E. Vitamin D3 (3a671), 45 mg vitamin E (3a700), 12 mg copper as copper (II) sulphate, pentahydrate (E4), 57 mg iron as iron carbonate (3b101), 60 mg zinc as zinc sulphate, monohydrate (3b605), 80 mg manganese as manganese (II) oxide (3b502), 1.2 mg iodine as calcium iodate, anhydrous (3b202), 0.35 mg selenium as sodium selenite (E8), 2.260 mg hydroxy analogue of methionine (min. 88% total acid).

**Technological additives:** 17.0 mg butylated hydroxytoluene (BHT) (E321), 7.0 mg Propyl gallate (E310), citric acid (E330), formic acid (E236), lactic acid (E270), propionic acid (E280).

**Coccidoistatic and histomonostatic agents:** 100 mg monensin sodium (51701).

**Poor diet:**

**Ingredients:** Crude protein 14.5%, crude fat 4.0%, crude fibre 5.0%, crude ash 6.0%, lysine 0.58%, methionine 0.30%, calcium 1.0%, phosphorus 0.6%, sodium 0.15%, energy 11.4 MJ ME/kg.

**Additives per kg:**

**Nutritional additives:** 8,000 i.U. Vitamin A (3a672a), 2.400 i.E. Vitamin D3 (3a671), 16 mg vitamin E (3a700), 12 mg copper as copper (II) sulphate, pentahydrate (E4), 57 mg iron as iron carbonate (3b101), 60 mg zinc as zinc sulphate, monohydrate (3b605), 80 mg manganese as manganese (II) oxide (3b502), 1.2 mg iodine as calcium iodate, anhydrous (3b202), 0.35 mg selenium as sodium selenite (E8), 1.100 mg hydroxy analogue of methionine (min. 88% total acid).

**Technological additives:** 17.0 mg butylated hydroxytoluene (BHT) (E321), 7.0 mg Propyl gallate (E310), citric acid (E330), formic acid (E236), lactic acid (E270), propionic acid (E280).

**Coccidoistatic and histomonostatic agents:** 100 mg monensin sodium (51701).


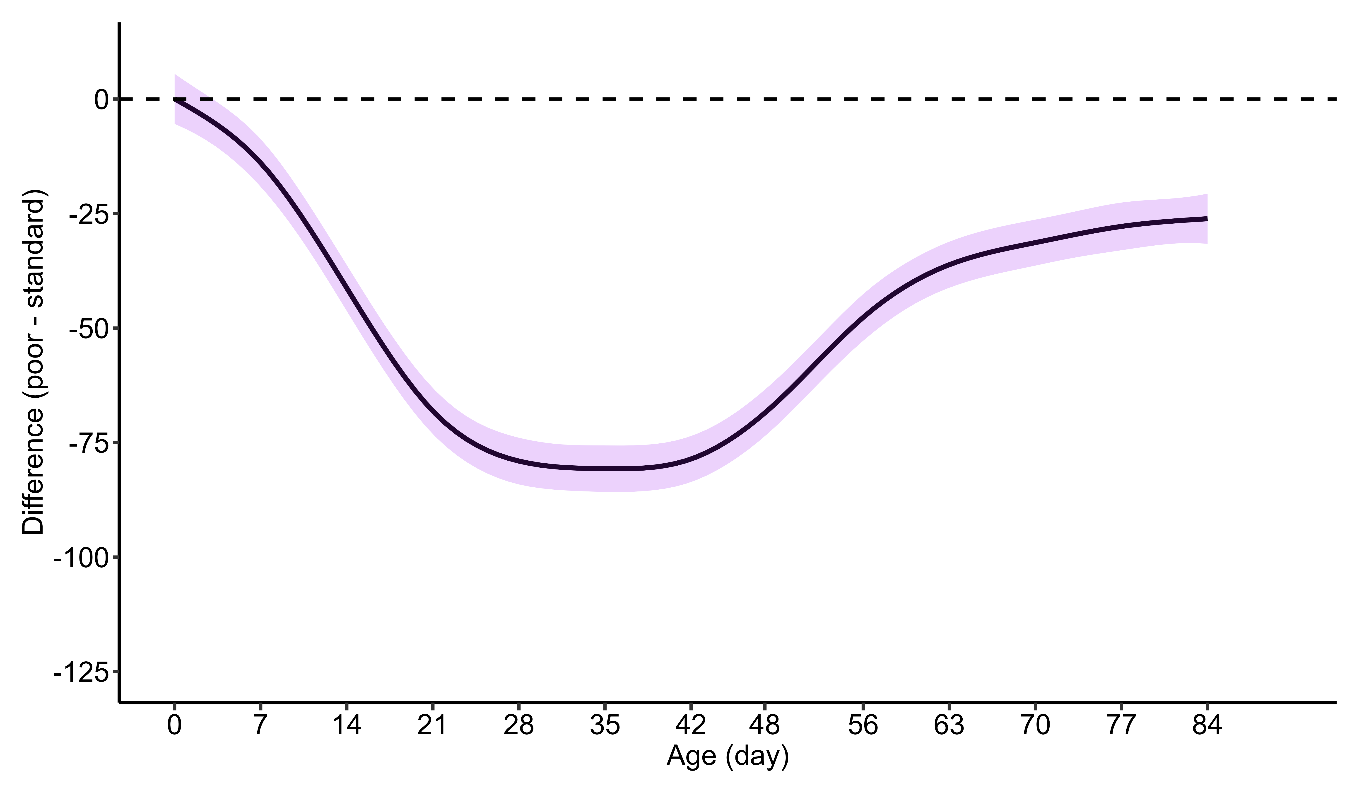


**Figure S1** Model predicted difference in body mass (in g), with 95% CI, between female Japanese quail chicks fed with a standard or poor rearing diet, in relation to age.

**
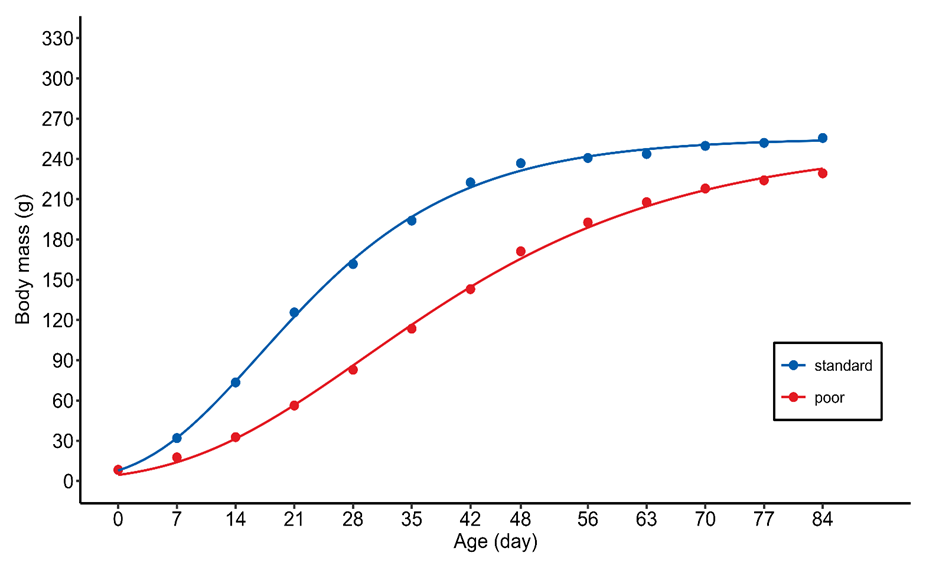
**

**Figure S2** Growth in body mass of female Japanese quail chicks fed with a standard or poor rearing diet. Solid lines represent model predictions of the Gompertz model (see Tables S1, S2). Datapoints represent the raw averages per measurement age.

| Model | AIC value |
| --- | --- |
| Gompertz | 25286.24 |
| Logistic | 25350.43 |
| Log-logistic | 25409.44 |
| Weibull | 25591.25 |

**Table S1.** AIC values of growth models using four common growth functions, ranked according to goodness of fit.

**Table S2.** Parameter values of the Gompertz growth function fitted for female Japanese quail chicks fed with a standard or poor rearing diet.

| Parameter | Estimate (± SE)  standard diet | Estimate (± SE)  poor diet |
| --- | --- | --- |
| Intrinsic growth constant (k) | 0.074 (± 0.001) | 0.047 (± 0.001) |
| Inflection point (t_i_, in days) | 16.844 (± 0.177) | 29.434 (± 0.350) |
| Asymptotic mass (A, in g) | 255.431 (± 1.137) | 251.150 (± 2.671) |

**Table S3.** Summary of a Weibull mortality model testing for effects of the rearing diet on age-specific adult mortality hazard of female Japanese quail.

|  | Estimate | Lower-95% | Upper-95% | SE |
| --- | --- | --- | --- | --- |
| Scale | 867.363 | 802.017 | 938.032 | 34.663 |
| Shape | 2.808 | 2.451 | 3.217 | 0.165 |
| Scale (poor) | -0.060 | -0.165 | 0.044 | 0.053 |
| Shape (poor) | -0.073 | -0.273 | 0.128 | 0.102 |
| Hatching year (2020) | -0.192 | -0.298 | -0.085 | 0.054 |

**Table S4** Summary of a LMM testing for an interaction effect between rearing diet and hatching year on adult lifespan (in days) of female Japanese quail.

| Random effects | Variance |  | Fixed effects | Estimate | SE | p-value | |
| --- | --- | --- | --- | --- | --- | --- | --- |
| Pair identity | 8794 |  | Intercept | 761.68 | 34.12 | <0.001 |  |
|  |  |  | Diet (poor) | -29.42 | 44.31 | 0.508 |  |
|  |  |  | Hatching year (2020) | -113.90 | 58.33 | 0.053 |  |
|  | |  | Diet : Hatching year | -32.34 | 82.52 | 0.696 |  |

**
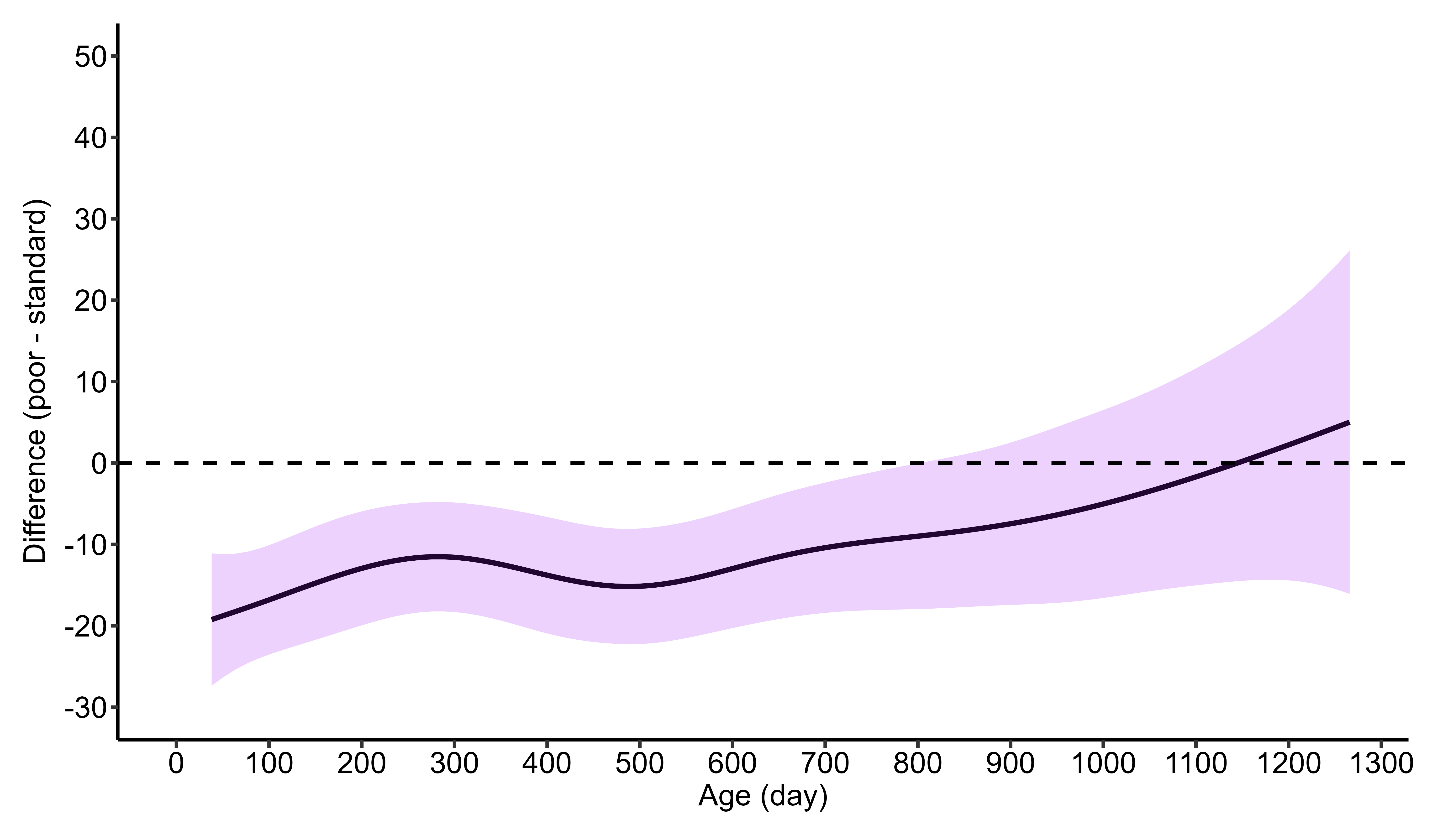
Figure S3** Model predicted difference in adult body mass (in g), with 95% CI, between female Japanese quail reared with a standard or poor diet, in relation to age.

**
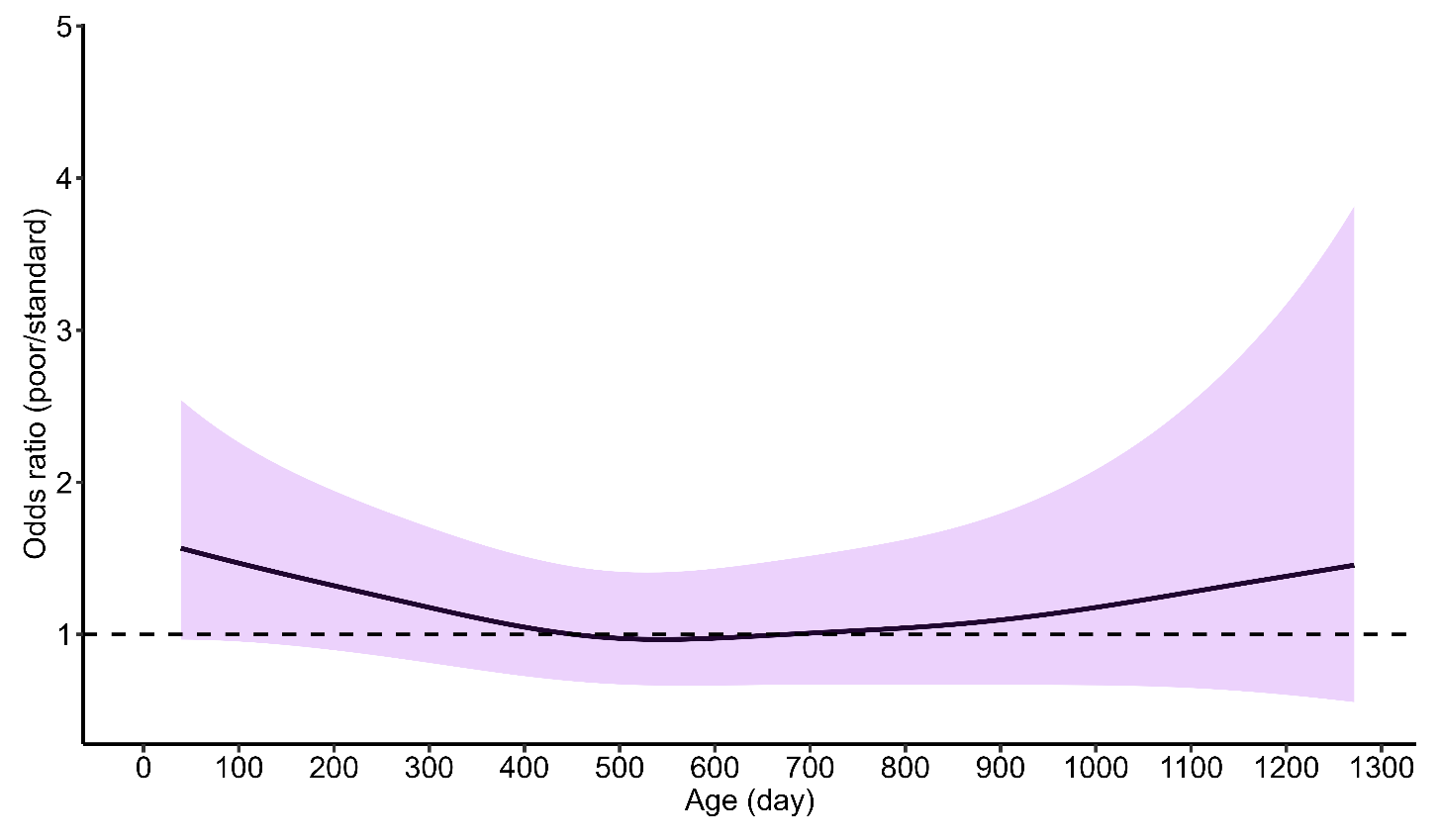
**

**Figure S4** Model predicted relative difference (odds ratio), with 95% CI, in daily laying probability of female Japanese quail reared with a standard or poor diet. The dashed line represents the odds ratio for which there is no difference between the standard and poor rearing diet.


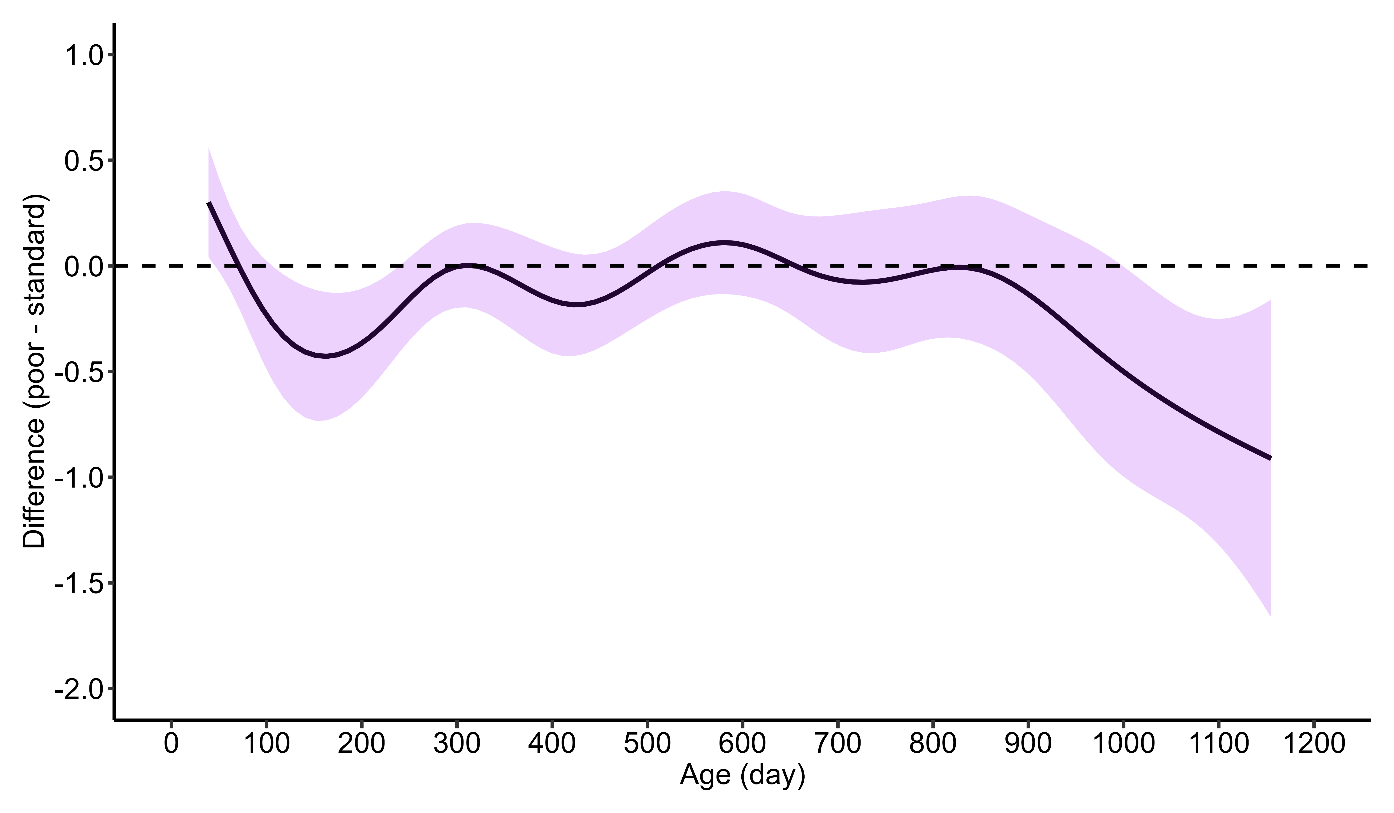


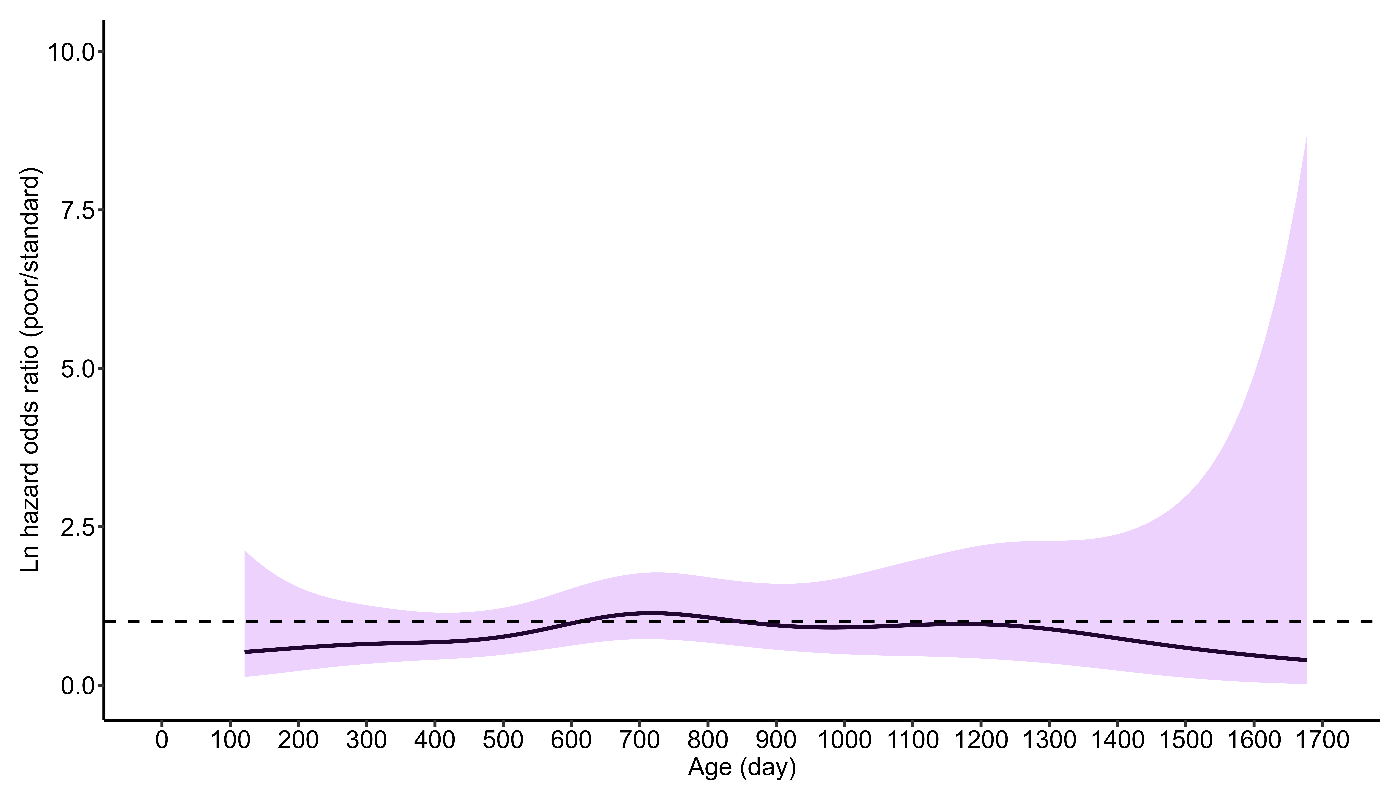
**Figure S5** Model predicted difference (in g), with 95% CI, in mass of eggs laid by female Japanese quail reared with a standard or poor diet, in relation to age.

**Figure S6** Model predicted relative difference (odds ratio), with 95% CI, in adult mortality hazard of female Japanese quail reared with a standard or poor diet, in relation to age. The dashed line represents the odds ratio for which there is no difference between the standard and poor rearing diet.

**Description of calculating the average lifetime number of eggs laid**


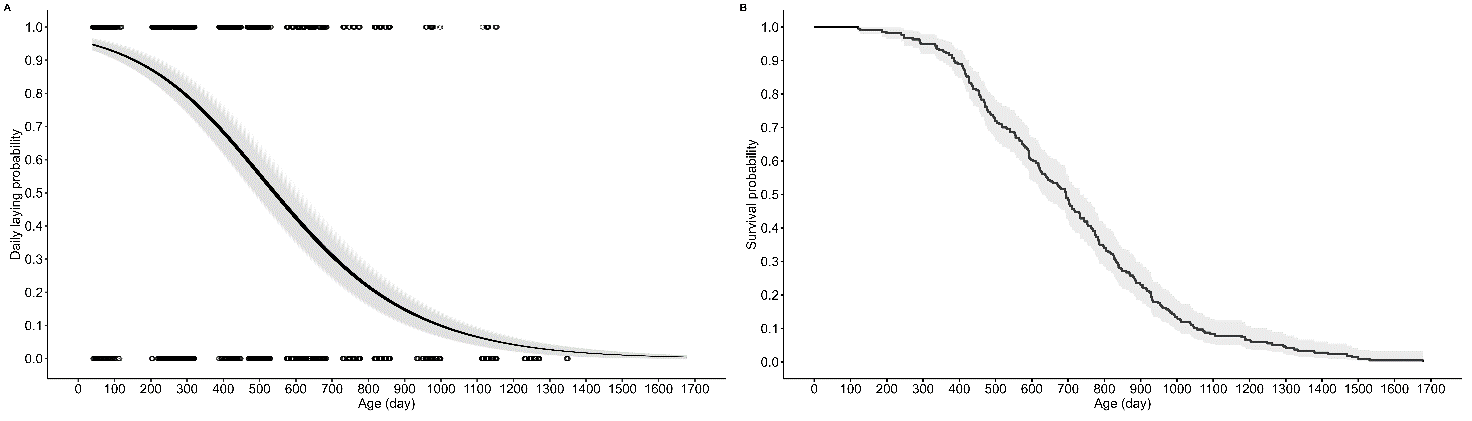
We estimated the average lifetime number of eggs laid by an adult female Japanese quail, under the conditions experienced in our study, by combining our detailed knowledge on age-specific laying rate and age-specific adult survival. Because the rearing diet had no detectable effects on age-specific laying rate we estimated the age-specific laying rate irrespective of the rearing diet. To this end, we ran a GAM (similar as in the main text, but without ‘diet’ added as a fixed effect) to obtain model predictions for the probability to lay an egg per age, in days. This allowed us to interpolate and extrapolate the laying probability to the ages at which we did not measure laying rate (see Fig. S7A). We subsequently multiplied this probability with the observed proportion of females reaching that age (irrespective of treatment), for every age (in days) until the maximum lifespan observed in our study (i.e., 1,678 days, see Fig. S7B). Summing up these values for every day after the start of laying will then give an estimate of the total lifetime number of eggs laid according to average age-specific laying and survival probabilities. Since females reared with the standard diet started to lay, on average, at an age of 55 days (main text; Table 2) this summed up to 395 eggs for these females. A 23 days later start of laying, as observed for the females reared with the poor diet (main text; Table 2), would then result in a reduction of 21 eggs (= 5%).

**Figure S7** A) Model predicted daily laying probability of female Japanese quail in relation to age, with 95% CI (shaded). The datapoints represent the raw data, and indicate the ages at which individuals were sampled. B) Observed probability of an adult female to reach a certain age, with 95% CI (shaded).
